# Involvement of p53-dependent apoptosis signal in antitumor effect of Colchicine on human papilloma virus (HPV)-positive human cervical cancer cells

**DOI:** 10.1042/BSR20194065

**Published:** 2020-03-25

**Authors:** Luchun Yan, Hao Huang, Ying Zhang, Xinrong Yuan, Zhaohua Yan, Chunyan Cao, Xiping Luo

**Affiliations:** 1 First Affiliated Hospital of Jinan University, Guangzhou 510632, P.R. China; 2 Department of Obstetrics and Gynecology, Affiliated Hospital of Guangdong Medical University, Zhanjiang 524001, P.R. China; 3 Department of Gynecology, the first Naval Hospital of Southern Theater Command, Zhanjiang 524005, P.R. China; 4 Department of Gynecology, Guangdong Women and Children Hospital, Guangzhou 511442, P.R. China

**Keywords:** Antitumor, Apoptosis, Colchicine, Human papillomavirus (HPV), p53

## Abstract

Colchicine, a plant-derived alkaloid with relatively low toxicity on normal human epidermal keratinocytes (HEKn), has selective inhibitory effect on the growth of CaSki (HPV16-positive) and HeLa (HPV18-positive) human cervical cancer cell lines via the induction of apoptosis. Colchicine (2.5, 5.0 and 10.0 ng/ml) significantly reduced the expression of human papilloma virus (HPV) 16 E6/E7 mRNA and protein in CaSki and HeLa cells. Moreover, reduced expression of E6 and E7 induced by Colchicine resulted in the up-regulation of tumor suppressor proteins, p53 and Rb, as well as down-regulation of phospho Rb (pRb) protein. In addition, Bax, cytosolic cytochrome *c* and cleaved caspase-3 protein were increased while Bcl-2 protein was decreased significantly by 48 h of Colchicine treatment. These results implied that Colchicine could be explored as a potent candidate agent for the treatment and prevention of HPV-associated cervical cancer without deleterious effects.

## Introduction

Cervical cancer is one of the most common malignancies all over the world and ranked the second leading causes of cancer-related death in females [1,2], which is closely related with persistent infection with oncogenic or high-risk (HR) HPV. It is well established that nearly over 90% of cervical cancers are caused by HR-HPV [3]. In 2000, an estimated, 468,000 new cases were diagnosed annually, of which about 80% occurred in developing countries [4], and such extensive screening programs make them inappropriate for the developing world with limited resources [5]. Current common treatment strategies for cervical cancer include surgery, radiotherapy and chemotherapy alone or in combination. However, 5-year overall survival rates with this treatment are about only 50% [6]. Although the availability of two HPV vaccines, Cervarix® or Gardasil®, offer prophylactic protection against a minor fraction of HPV types-associated cervical lesions, they are not effective for existing post-HPV infection lesions [7,8]. What’s more, the cost of vaccination programs makes them inappropriate for developing countries. Despite advances in prevention and treatment of this disease, protocols for recurrence in a refractory form and available treatment options without undesirable side-effects are scanty. In recent years, more effective and less toxic anti-cancer agents from natural resources have become a research hot topic for cancer prevention and treatment based on their ability to attack multiple molecular targets [9]. These highlight an urgent need for development of efficacious plant-derived virus-specific inhibitors to overcome HPV-associated cervical cancer.

Colchicine is a very cheap natural alkaloid derived from either *Colchicum autumnale* (meadow saffron) or *Gloriosa superba* (glory lily) that has been utilized in the treatment of gout since 200 years ago [10,11]. It is an antimitotic compound, which has very strong binding capacity to β-tubulin heterodimers in solution to perturb microtubule dynamics, thus leading to cell cycle arrest, apoptosis and cell death [12]. Such inhibition of microtubule formation may contribute to the establishment of improved cancer therapies because cancer cells proliferate rapidly and uncontrollably [13,14]. Latter studies suggested that colchicine has notable effects in cancer cells including cervical cancer [15], which implies that colchicine may have potential as an anti-cancer drug for cervical cancer. Although colchicine exerts potent antimitotic properties, its mechanism of inducing cervical cancer cell apoptosis remains unclear. Understanding the anticancer mechanism of colchicine may lead to new clinical applications in cervical cancer treatment. Therefore, the purpose of the present study was to investigate whether colchicine also had anticancer effects on cervical cancer cells and its possible molecular mechanisms.

## Materials and methods

### Materials and chemicals

Purified Colchicine (99.89%), MTT, and DMSO were purchased from Sigma–Aldrich Co. (St. Louis, MO, U.S.A.). Antibodies against Bcl-2, Bax, cleaved-caspase-3, p53, Rb, phospho Rb (pRb), β-actin and horseradish peroxidase (HRP) secondary conjugated antibodies were obtained from Santa Cruz Biotechnology, Inc., (Santa Cruz, CA, U.S.A.). Dulbecco’s Modified Eagle’s Medium (DMEM), RPMI 1640, fetal bovine serum (FBS), penicillin, and streptomycin were obtained from Cell Signaling Technology (Danvers, MA, U.S.A.). Annexin V-fluorescein isothiocyanate (FITC) apoptosis detection kit and propidium iodide (PI) were purchased from BD Bioscience (San Jose, CA, U.S.A.).

### Cell culture

The human cervical cancer cell lines CaSki (HPV 16 positive), HeLa (HPV 18 positive), C-33A (HPV negative) and primary human epidermal keratinocytes (HEKn, normal) were obtained from the Cell Bank of Shanghai Institute of Biochemistry and Cell Biology, Chinese Academy of Sciences (Shanghai, PR China) and grown according to their specifications. Cervical cancer cells were cultured in Dulbecco’s modified Eagle’s medium or RPMI 1640 media supplemented with 10% (v/v) heat-inactivated FBS, 100 U/ml penicillin G and 100 mg/ml streptomycin in a humidified atmosphere containing 95% air and 5% CO_2_ at 37°C. Primary HEKn cells were cultured in basal cell dermal media supplemented with keratinocyte growth kit components and antibiotics, as per conditioned described above.

### Cytotoxicity assay

The cytotoxic effects of Colchicine on cell viability were determined with MTT assay [16]. Briefly, cells (10,000 per well) were seeded into 96-well culture plates and incubated with different concentrations of Colchicine for varying period. Subsequently, 20 μl MTT (5 mg/ml) reagents were added into each well containing the untreated and treated cells and incubated at 37°C for another 4 h, followed by the addition of 10 μl DMSO to dissolve the formazan. Then, the absorbance values were measured at 490 nm on an automatic microplate reader (Bio-Rad Benchmark, California, U.S.A.). The effect of Colchicine on growth inhibition was defined as percentage of cell viability, where cells with no treatment were considered 100% viable. Morphological changes in treated or untreated cells were observed using a phase-contrast inverse microscope (Olympus, Japan).

### Annexin V/propidium iodide assay for apoptosis

Apoptosis was detected using an annexin V-FITC apoptosis detection kit according to the manufacturer’s instructions. In brief, treated or untreated cells were harvested, washed twice with cold phosphate-buffered saline, and then stained with 10 μl Annexin V- FITC and 10 μl of PI (20 μg/ml) in the dark at room temperature for 20 min. Apoptotic cells were analyzed immediately by BD FACScan flow cytometer (Becton Dickinson, San Jose, CA) with CellQuest 3.0 software. For each sample, the fluorescence of 10,000 cells was gated and counted. The degree of apoptosis was quantified as a percentage of the annexin V positive and PI-negative (annexin V^+^/PI^−^) cells [17].

### Quantitative real-time reverse transcription PCR (qRT-PCR) analysis of HPV16 E6 and E7 transcripts

Following different treatment, both adherent and floating cells were collected and frozen at −80°C until analysis. Total cellular RNAs were isolated with TRIzol reagent as per manufacturer’s protocol. RNA concentration and purity were evaluated on an ultraviolet spectrophotometer (Bio-Rad Inc., Hercules, CA, U.S.A.) by measuring the ratio of absorbance at 260 and 280 nm. qRT-PCR for the HPV E6, E7 mRNA and internal standard β-actin were performed on an ABI 7500 Fast RT-PCR system (Applied Biosystems, Foster City, CA, U.S.A.) using SuperScript III Platinum SYBR Green One-step RT-PCR Kit (Invitrogen, Carlsbad, California, U.S.A.) with the following procedures: 50°C for 4 min, 95°C for 14 min, followed by 40 cycles of 95°C for 15 s and 60°C for 31s. The primer sequences were as list below: HPV16 E6, forward: 5′-CTGCAATGTTTCAGGACCCA-3′ and reverse: 5′-TCATGTATAGTTGTGCAGCTCTGT-3′; HPV16 E7, forward: 5′-GAGGA GGAGGATGAAATAGATGGT-3′ and reverse: 5′-CACTTGCAACAAAACGTT ACAATATTG-3′; HPV18 E6, forward: 5′-AAGATTTATTTGTGGTGT-3′ and reverse: 5′-GGTGGATTG-3′; HPV18 E7, forward: 5′-CACGTAGAGAAACCCAGCTGTAA-3′ and reverse: 5′-GCAGGATCAGCCATGGTAGATT-3′; β-actin, forward: 5′- CCAACCGCGAGAAGATGA-3′ and reverse: 5′- CCAGAGGCGTACAGGGATAG-3′. Each measurement was performed in triplicate, and no-template controls were included for each assay. After completion of PCR, a dissociation curve analysis was done and *C*t values were obtained from the ABI 7500 fast v2.0.1 software. Relative mRNA fold change was calculated using the 2^−ΔΔ^CT method [18].

### Protein extraction and Western blot analysis

Cells with indicated treatments were lysed with ice-cold lysis buffer (50 mM Tris-HCl, 150 mM NaCl, 1% NP40, 100 mM DTT, 0.1% SDS and 10% glycerol). The protein concentration was quantified using the BCA method. A total of 25 μg protein was separated using 10–15% SDS-PAGE and then transferred onto polyvinylidene fluoride membranes. The membrane was blocked with 5% nonfat milk in TBST solution and incubated overnight at 4°C with primary antibodies against Bcl-2 (1:1000), Bax (1:1000), cleaved-caspase-3 (1:500), p53 (1:1,000), Rb (1:1000), pRb (1:1000) and β-actin (1:2000), followed by incubation with HRP-conjugated second antibodies. Protein bands were visualized using the enhanced chemiluminescence detection system (ECL, Amersham Biosciences, Buckinghamshire, U.K.). The relative protein levels were calculated based on β-actin protein as a loading control.

### Statistical analysis

Results are expressed as mean of three individual experiments ± standard deviation (SD), and data were analyzed using the GraphPad Prism version 6.01 statistical program (GraphPad Software, Inc.). Statistical differences between groups were evaluated using one-way analysis of variance (ANOVA) followed by the Student’s *t*-tests. *P* < 0.05 was considered to indicate a statistically significant result.

## Results

### Cytotoxicity of Colchicine on CaSki, HeLa, C-33A and HEKn cells

**Figure 1:**
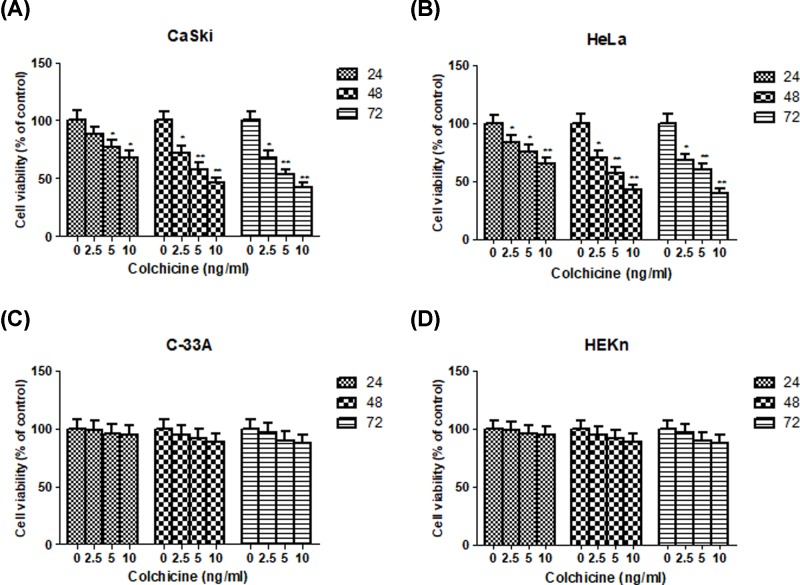
Cytotoxicity of Colchicine on CaSki, HeLa, C-33A and HEKn cells (**A**) The effect of Colchicine on cell viability of CaSki cells. (**B**) The effect of Colchicine on cell viability of HeLa cells. (**C**) The effect of Colchicine on cell viability of C-33A cells. (**D**) The effect of Colchicine on cell viability of HEKn cells. Values are expressed as mean ± S.D. of three independent experiments. *P <0.05 and **P <0.01 compared with control.

To determine the cytotoxic effect of Colchicine on cell growth of different HPV cervical cancer cell lines, CaSki (HPV 16 positive), HeLa (HPV 18 positive), C-33A (HPV negative) and normal HEKn cells exposed to increasing doses of Colchicine for 24, 48 and 72 h, and cell viability was examied by the MTT assay. The results showed that the cell viabilities of CaSki and HeLa were markedly decreased after exposure to Colchicine in dose- and time-responsive manners. It was observed that Colchicine decreased the growth of CaSki cells by 12.21% (2.5 ng/ml), 23.02% (5.0 ng/ml, *P* < 0.05) and 32.25% (10.0 ng/ml, *P* < 0.05) at 24 h, by 28.21% (2.5 ng/ml, *P* < 0.05), 42.62% (5.0 ng/ml, *P* < 0.01) and 54.12% (10.0 ng/ml, *P* < 0.01) at 48 h, and by 32.50% (2.5 ng/ml, *P* < 0.05), 47.14% (5.0 ng/ml, *P* < 0.01) and 58.25% (10.0 ng/ml, *P* < 0.01) at 72 h, respectively, compared with the untreated control cells (Figure 1A). Similarly, HeLa cells exhibited, 17.34% (2.5 ng/ml, *P* < 0.05), 25.05% (5.0 ng/ml, *P* < 0.05) and 34.98% (10.0 ng/ml, *P* < 0.01) decrease in the cell growth at 24 h, 30.25% (2.5 ng/ml, *P* < 0.05), 43.21% (5.0 ng/ml, *P* < 0.01) and 57.02% (10.0 ng/ml, *P* < 0.01) decrease at 48 h, and 32.68% (2.5 ng/ml, *P* < 0.05), 40.37% (5.0 ng/ml, *P* < 0.01) and 59.67% (10.0 ng/ml, *P* < 0.01) decrease at 72 h, respectively, compared with the untreated control cells (Figure 1B). We observed that the inhibitory effect of Colchicine on cell proliferation of CaSki or HeLa cells at 48 h reached the same potent efficiency at 72 h, thus we choose the 48-h incubation time as following assay. Nevertheless, no cell viability inhibitory effect was observed in C-33A cervical cancer cells and HEKn normal cells following Colchicine treatment in the same condition (Figure 1C,D). This result indicated that Colchicine exhibited selective toxicity for HPV positive CaSki and HeLa cervical cancer cells.

### Effects of Colchicine on the apoptosis in CaSki and HeLa cells

**Figure 2:**
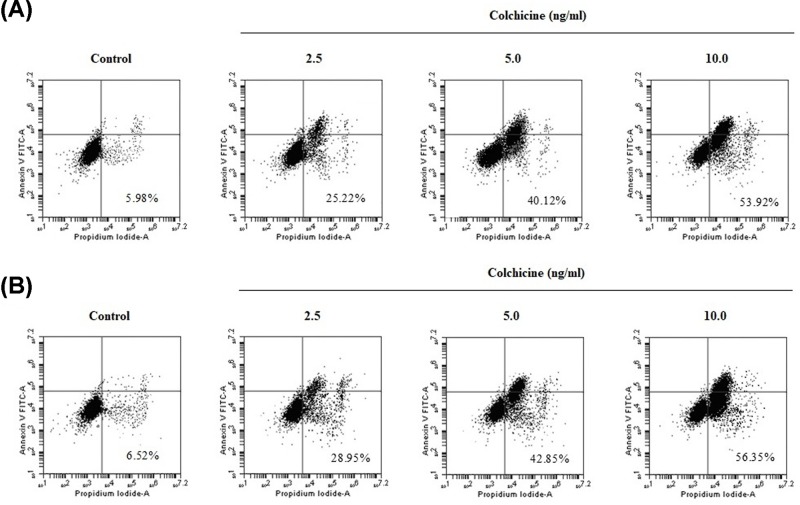
Effects of Colchicine on the apoptosis in CaSki and HeLa cells (**A**) The effect of Colchicine on apoptosis of CaSki cells. (**B**) The effect of Colchicine on apoptosis of HeLa cells. Values are expressed as mean ± S.D. of three independent experiments.

To determin whether Colchicine induced inhibition of cell proliferation is related to the induction of apoptosis, an annexin V-FITC apoptosis detection kit was used to quantify the rate of apoptosis in both cervical cancer cells. Flow cytometry revealed that the number of apoptotic cells was increased in response to treatment with Colchicine by 25.22% (2.5 ng/ml), 40.12% (5.0 ng/ml) and 53.92% (10.0 ng/ml) for CaSki cells, and 28.95% (2.5 ng/ml), 42.85% (5.0 ng/ml) and 56.35% (10.0 ng/ml) for HeLa cells, as compared with the control group, respectively (Figure 2A,B). Therefore, the antiproliferation activity of Colchicine on CaSki or HeLa cells was related to apoptosis.

### Effect of Colchicine on the expression of HPV E6 and E7 in CaSki and HeLa cells

**Figure 3:**
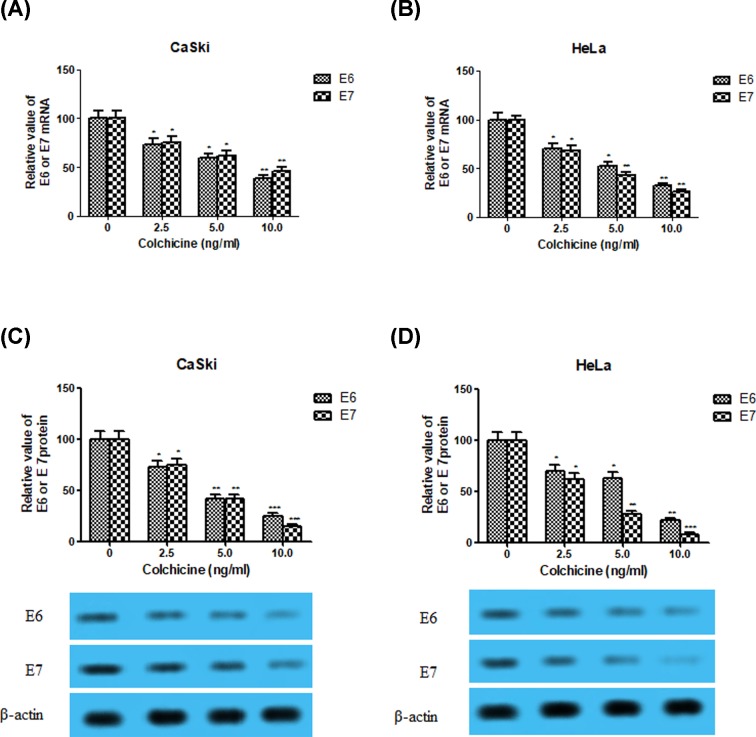
Effect of Colchicine on the expression of HPV E6 and E7 in CaSki and HeLa cells (**A**) The effect of Colchicine on the mRNA expression of E6 and E7 in CaSki cells. (**B**) The effect of Colchicine on the mRNA expression of E6 and E7 in HeLa cells. (**C**) The effect of Colchicine on the protein expression of E6 and E7 in CaSki cells. (**D**) The effect of Colchicine on the protein expression of E6 and E7 in HeLa cells. Values are expressed as mean ± S.D. of three independent experiments. *P <0.05, **P <0.01 and ***P <0.001 compared with control.

Colchicine exhibited significant sensitive toxicity to both HPV16 (CaSki) and HPV18 (HeLa) positive cell lines via induction of apoptosis, so next we aimed to investigate the expression of the viral E6 and E7 gene or proteins in two cell lines by qRT-PCR or Western blot, respectively. There was a significant dose-dependent reduction of E6 and E7 mRNA or oncoproteins in both the cervical cancer cell lines, CaSki (Figure 3A,C) and HeLa (Figure 3B,D), compared with those in the untreated control cells (*P* < 0.05, *P* < 0.01 or *P* < 0.001). All these results suggested decreased the expression of the HPV E6 and E7 were involved in the antitumor effect of Colchicine toward CaSki and HeLa cells.

### Effects of Colchicine on the expression of p53, Rb and pRb proteins

**Figure 4:**
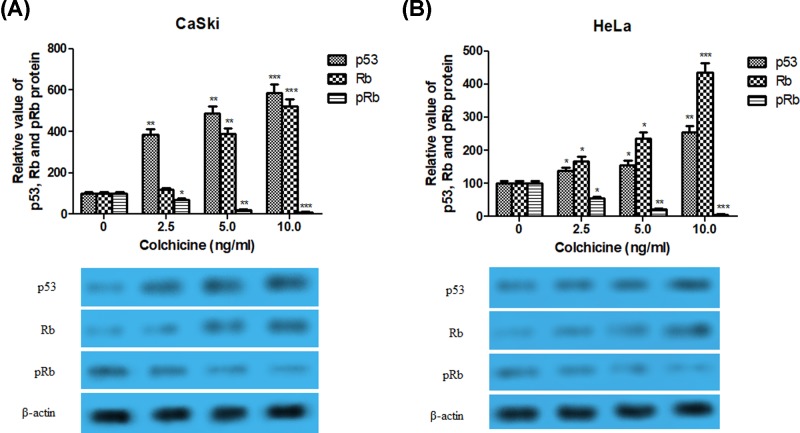
Effects of Colchicine on the expression of p53, Rb and pRb proteins in CaSki and HeLa cells (**A**) The effect of Colchicine on the protein expression of p53, Rb and pRb in CaSki cells. (**B**) The effect of Colchicine on the protein expression of p53, Rb and pRb in HeLa cells. Values are expressed as mean ± S.D. of three independent experiments. *P <0.05, **P <0.01 and ***P <0.001 compared with control.

Considering the repression of oncoproteins E6 and E7, we thus examined the effect of Colchicine on the expression of p53, Rb and pRb protiens in both cervical cancer cells. The results showed that Colchicine dose-dependently up-regulated p53 and Rb protein expressions in HPV-positive CaSki cells or HeLa cells (Figure 4A,B). In contrast, the expression of pRb was decreased dose dependently in both cancer cells. Interestingly, no change was observed on p53 and Rb proteins level in HPV-negtive C-33A cells (data not shown). These results confirmed that reduced expression of E6 and E7 is the key contributor to Colchicine-mediated up-regulation of p53 and Rb.

### Effects of Colchicine on apoptosis-related protein in CaSki and HeLa cells

**Figure 5:**
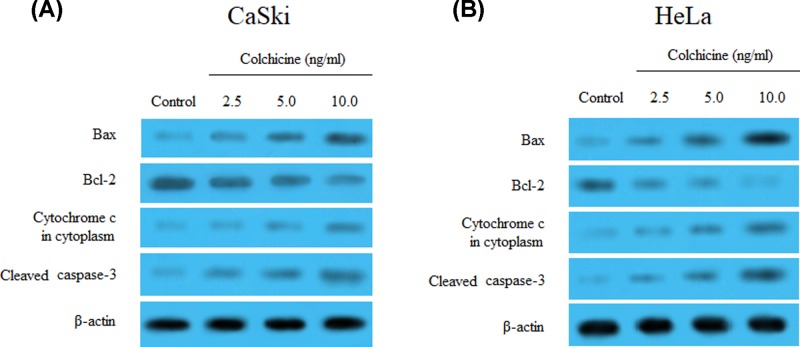
Effects of Colchicine on apoptosis-related protein in CaSki and HeLa cells (**A**) The effect of Colchicine on the protein expression of Bax, Bcl-2, cleaved caspase 3 and cytoplasmic cytochrome *c* in CaSki cells. (**B**) The effect of Colchicine on the protein expression of Bax, Bcl-2, cleaved caspase 3 and cytoplasmic cytochrome *c* in HeLa cells.

To further explore the effects of Colchicine on the apoptosis of CaSki and HeLa cells, the present study determined the altered expression levels of apoptosis-associated proteins by Western blotting. When both cervical cancer cells were treated with 2.5, 5.0 and 10.0 ng/ml of Colchicine for 48 h, the protein expression levels of Bax, cleaved caspase 3 and cytochrome *c* in cytosolic fraction were increased in a dose-dependent manner, whereas the Bcl-2 protein expression was in the opposite manner (Figure 5A,B).

## Discussion

During cervical carcinogenesis, an increasing imbalance between proliferation and apoptosis (programmed cell death) has been observed, when compared with normal cervical epithelium. HPV is the major cause of development of cervical cancer. As the prevalence of HPV infection increased, the incidence of cervical cancer increased rapidly [19,20]. HPVs included many subtype, such as HPV 16, 18, 31 and 33, out of which HPV-16 and HPV-18 are the most common genotypes in malignant biopsies with little geographic variation and account for almost 70% of the cancers [21,22]. Two HPV encoded viral oncoproteins, E6 and E7, are essential for cervical carcinogenesis by their potential to inactivate the tumor suppressor proteins p53 and retinoblastoma protein (pRb), respectively [23]. These tumor suppressor proteins are involved in many signaling pathways that regulate the cell cycle and monitor and protect the integrity of the genome [24,25]. The anti-oncogene p53 has long been involved in tumor necrosis factor (TNF)-α-mediated apoptosis, which is achieved by induction of cell cycle G1 arrest and inhibition of cell proliferation, thus leading eventually to apoptosis and cell death [26,27]. Despite the critical role of p53 in cell survival, characterization of the downstream signaling events remains largely undefined at present. As of now, there is a cross-talk between the p53 and TNF-α mediated pathway. Once transcription factor NF-κB-induced threshold level of p53 is reached as a result of TNF-α stimulation, the cells will be definitely committed to apoptosis [27–29]. Moreover, continual cell stress induced by TNF-α may actually lead to up-regulation of p53 and the inactivation of IκB kinase 2 (IKK2 or IKKβ), which is responsible for triggering the nuclear import of the NF-κB [26,30]. Thereof, the resulting NF-κB suppression is able to inactivate the transcription of genes that regulate a variety of fundamental biological processes, including apoptosis [30]. Unlike other kind of cancers, where the p53 gene is mutated irreversibly, cervical cancer cell lines uniquely harbor wild-type p53 and Rb genes [31]. In this case, the growth-regulatory machinery of normal cells is masked by the expression of HPV E6 and E7 proteins [32]. To this end, HPV E6 and E7 oncoproteins have become a target for cervical cancer prevention and treatment. Although HPV vaccines against two main stains of HPV (16 and 18) by large pharmaceutical companies block the development of cervical cancer in a great degree, an estimated cost at $300 for a person in vaccination programs will prohibit the access of most women in developing countries to obtain these vaccines [33]. Down-regulation of viral HPV E6 and E7 represents a viable strategy to induce functional p53 for cervical cancer therapies, which would in turn further induce downstream target genes involved in cell circle arrest and apoptosis and thus restore growth control in tumor cells or sensitize cells to cancer therapies [34]. Actually, consistent with this notion, there has been a growing body of evidence to suggest that the possibility of reactivating the p53 pathway has been extensively studied in several cancers [35,36].

In our screening for drug candidates exhibiting inhibitory activity against HPV, we found that Colchicine displayed dramatic growth inhibitory effect on HPV-16 (CaSki) or -18 (HeLa) positive cervical cancer cell lines, but not on HPV negative (C-33A) cell line. The antiproliferative effect of Colchicine was clearly associated with induction of apoptosis, as evidenced by the increasing percentage of apoptotic CaSki or HeLa cells after Annexin/PI staining on flow cytometer. These results showed that CaSki and HeLa cells treated with Colchicine undergo typical apoptosis. However, these changes did not occur in HPV negative C-33A and normal HEKn cells. Furthermore, RT-PCR and Western blotting indicated that Colchicine dose-dependently inhibited both mRNA and proteins expression of HPV E6 and E7 in CaSki and HeLa cells, respectively, confirming that Colchicine might have potential inhibitory activity against cervical cancer associated with HPV. We next examined if repression of HPV E6 and E7 oncogenes resulted in reactivation of dormant tumor suppressor p53 and Rb. It was evident that Colchicine dose-dependently increased the expression of p53 and Rb tumor suppressor proteins in CaSki and HeLa cells, but decreased pRb protein expression. At the same time, the tumor suppressor protein levels in the HPV negative cell lines C-33A largely remained unaltered. Hence, we presumed that Colchicine did not directly change p53 and Rb expression, and the resulting up-regulation in both cancer cells was caused by the down-regulation of E6 and E7.

The restoration of tumor suppressor functions of p53 proteins might lead to the activation of downstream signaling molecules, such as Bax, Bcl-2, cytochrome *c* and cleaved caspase-3, thus finally causing either cell cycle arrest or apoptosis [37]. The change of these apoptosis-related proteins is regulated by p53 via the protein–protein interaction and it is known as the intrinsic apoptotic pathway or mitochondrial-mediated pathway [38–40]. Western blotting indicated that the addition of Colchicine stimulated the protein expression of Bax, cytochrome *c* and cleaved caspase-3, but inhibited the expression of Bcl-2 protein in CaSki and HeLa cells. These results suggested that the antitumor effect of Colchicine on CaSki and HeLa cells was associated with the p53-dependent intrinsic apoptotic pathway.

## Conclusion

In summary, the present study demonstrated that Colchicine has profound *in vitro* antiproliferative activity against HPV-16 or -18 positive human cervical cancer cell lines, which was achieved by HPV E6/E7 inhibition and subsequently p53-dependent intrinsic apoptosis. Our findings suggest Colchicine may be exploited as a promising candidate agent for HPV associated cervical cancer prevention and treatment. Further more work including *in vivo* studies are required to further elucidate the exact mechanism and therapeutic effects.

## Abbreviations

HEKn: human epidermal keratinocytes
HPV: human papilloma virus
pRb: phospho Rb
TNF: tumor necrosis factor
